# Clinical Implementation and Evaluation of Three Implementation Interventions for a Family-Oriented Care for Children of Mentally Ill Parents (ci-chimps): Study Protocol for a Randomized Controlled Multicenter Trial

**DOI:** 10.3389/fpsyt.2022.823186

**Published:** 2022-02-28

**Authors:** Carolin Laser, Anna Modarressi, Bjørg Eva Skogøy, Andrea Reupert, Anne Daubmann, Alexandra Höller, Antonia Zapf, Silke Pawils, Svenja Taubner, Sibylle Winter, Darryl Maybery, Silke Wiegand-Grefe

**Affiliations:** ^1^Department of Child and Adolescent Psychiatry and Psychotherapy, University Medical Centre Hamburg-Eppendorf, Hamburg, Germany; ^2^Nordland Hospital Trust, Nordland Research Institute, Bodø, Norway; ^3^Krongold Clinic, Faculty of Education, Monash University, Clayton, VIC, Australia; ^4^Department of Medical Biometry and Epidemiology, University Medical Centre Hamburg–Eppendorf, Hamburg, Germany; ^5^Institute and Outpatients Clinic of Medical Psychology, Centre for Psychosocial Medicine, University Medical Centre Hamburg-Eppendorf, Hamburg, Germany; ^6^Center for Psychosocial Medicine, Institute for Psychosocial Prevention, University Hospital Heidelberg, Heidelberg, Germany; ^7^Department of Child and Adolescent Psychiatry, Psychosomatics and Psychotherapy, Charité Berlin, Berlin, Germany; ^8^Department of Rural Health, Monash University, Clayton, VIC, Australia

**Keywords:** children of mentally ill parents, implementation research, family implementation interventions, randomized controlled trial, multicenter trial

## Abstract

**Background:**

In Germany, approximately three million children under the age of eighteen have a mentally ill parent. These children are at an increased risk of developing a mental illness themselves (1) as well as a physical illness (2). While research has identified numerous evidence-based family-oriented interventions, little is known about how to implement such interventions effectively and efficiently in clinical practice in Germany. This implementation study (ci-chimps) evaluates three clinical implementation projects with three different implementation interventions for the optimal implementation of the tailored family-oriented preventive and therapeutic interventions in the CHIMPS-NET (children of mentally ill parents—research network) with an implementation model for children of mentally ill parents.

**Methods:**

A two-group randomized controlled multicenter trial will examine changes in family-oriented practice and aspects of implementation at baseline as well as at 12- and 24-months follow-up. The CHIMPS-Network consists of 20 clinical centers. The centers in the intervention group receive the support of all of the three implementation interventions: (1) optimal pathways to care, (2) education and a training program for professionals, and (3) systematic screening for children. The centers in the control group do not receive this specific implementation support.

**Discussion:**

While we know that children of mentally ill parents are an important target group to be addressed by preventive and therapeutic interventions, there is often a lack of structured implementation of family-oriented interventions in clinical practice in Germany. Using a randomized controlled multicenter trial design with a large and wide-ranging sample (clinics for adult psychiatry and clinics for child and adolescent psychiatry, university clinics and clinics at the real health care) will provide a robust understanding of implementing family-oriented changes in German clinical practice.

**Trial Registration:**

The CHIMPS-NET-study was registered with the German Clinical Trials Register on 2019-12-19 (DRKS00020380) and with Clinical Trials on 2020-4-30 (NCT04369625), the ci-chimps-study was registered with the German Clinical Trials Register (DRKS00026217) on 2021-08-27, the Clinical Trials registration is in review process.

## Background

In Germany, approximately three million children under the age of 18 have a mentally ill parent (1). These children are at an increased risk of developing a mental illness themselves (1) as well as a physical illness (2). Not all children will be impacted in the same way, and children's outcomes vary depending on the severity and chronicity of the parents' illness, the support provided to the family, environmental factors (such as poverty and housing) and the timing of the illness in relation to the child's age (references see below) (1). To mitigate mental symptoms and concomitant diseases in these children and adolescents, Wiegand-Grefe et al. developed a low-frequency family-oriented intervention for children of mentally ill parents: CHIMPS (3). The term CHIMPS stands for “Children of mentally ill parents.” The acronym CHIMPS might be considered stigmatizing however we have used it positively by employing a chimpanzee as our project mascot. We argue that this makes the acronym fun and playful and thus engaging to children. The CHIMPS intervention has been manualized (3) and evaluated in waiting-list-controlled pilot studies (4–7), which indicates improvements in the mental health of the children (4), the health-related quality of life and social support of children and their families (5), the family functioning (6) and congruent and successful parental coping strategies (7). Furthermore, in the next step, this CHIMPS intervention program has been evaluated in a BMBF funded, multicenter trial (8).

In the current “Children of mentally ill parents-research network” (CHIMPS-NET), funded by the innovation fund at the GB-A, the CHIMPS intervention where updated and more adapted to the heterogeneous needs of each family. In CHIMPS-NET, in a stepped care model, every family with a mentally ill parent and with children from age 3–18 years is screened regarding his/her family functioning and the mental health state of his/her children and adolescents and the parents and allocated to different interventions according to his/her indication and the requirements. For further information about the different family-oriented preventive and therapeutic interventions in CHIMPS-NET, for the design of the evaluation studies, as well for the inclusion and exclusion criteria to each intervention see the study protocol of the central project CHIMPS-NET (9). This publication focused the implementation interventions and the design of the overarching implementation study ci-chimps.

### Implementation Research for Interventions for Children of Mentally Ill Parents

Because of the knowledge and the increasing awareness that children of mentally ill parents are an important target group to be addressed by preventive interventions, there are numerous programs and tools (10, 11). But there is often a lack of structured implementation of family-oriented interventions in clinical practice not only in Germany (10–13). Implementation can be defined as a specified set of activities designed to put into practice an activity or program of known dimensions (14). Lauritzen et al. describe three general implementation categories: “paper implementation,” “process implementation,” and “performance implementation” (13). Paper implementation puts new policies and procedures into place but does not change practice in itself. Process implementation incorporates new procedures into an organization, and performance implementation provides content and tools to practitioners, so that new procedures and processes have functional components for change. Ci-chimps belongs to the performance implementations by giving the clinical centers new tools like the screening (15).

Personal attitudes like self-reported skills and knowledge, beliefs about job role, and perceptions of workplace support seem to have a notable impact in supporting a successful and sustainable implementation (11, 16–20). Furthermore, organizational factors, such as reporting systems, meeting structures, leadership and supervision, are closely associated with satisfaction with the implementation process (11, 17, 19–21).

For family-oriented practice, the following barriers are reported: organizational barriers [policies, leadership and management (22–24)], high workload (22–24), patient-oriented treatment (24), no routines in identifying affected families (21–23), and gaps in mental health professionals' knowledge, and skills about children of mentally ill parents (21–23).

Gregg et al. summarized in their review factors influencing family-oriented practice in a detailed overall diagram with two main parts: Practitioner factors and workplace factors. They subclassify practitioner factors in personal attitudes like beliefs about family-oriented practice and professional subfactors like training and education, job role, skills and knowledge. Workplace factors were subclassified in service-related subfactors e.g., available resources and work setting and support-related subfactors like workplace support and time and workload (11).

Maybery and Reupert are providing an overview of the barriers regarding family-oriented practice. They have designed a model based on a hierarchy with the main factors “organizational policies and procedures (including managerial support),” “workers attitude, knowledge and skills,” and “barriers families themselves bring in” (22). Lauritzen and Reedtz adapted this model for Norway and added the two factors “organization of mental health care services” and “geographical conditions” (13). These papers present the international state of knowledge of the implementation of interventions especially for this target group of children of mentally ill parents.

### The Implementation Model of the CHIMPS-NET Consortium

**Figure 1:**
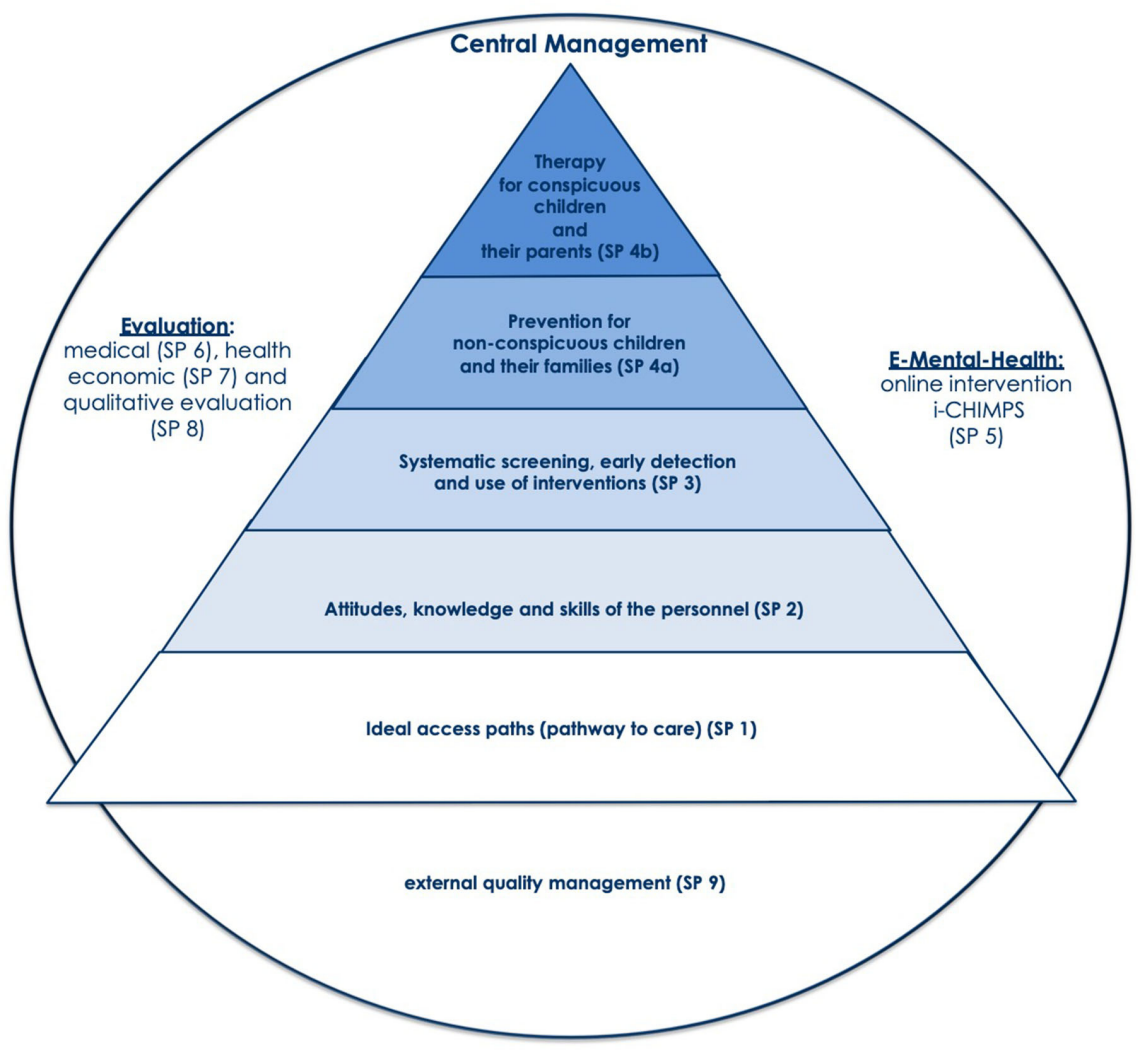
The CHIMPS-NET Implementation Model [based on (22) and first published (25)] for the health care for children and their mentally ill parents.

The implementation science showed that evidence-based interventions will not be effective if not properly implemented (19). In order to achieve a successful implementation, all CHIMPS interventions are based on the Australian model by Maybery and Reupert (22) and the Norwegian model by Lauritzen and Reedtz (13). The CHIMPS-NET consortium specifies the content of prevention and care according to the German healthcare system setting, including adult psychiatry and child and adolescent psychiatry, and develops an own implementation process based on three implementation interventions. Figure 1 shows the three subprojects (SP) for implementing and realizing the hierarchical components of an evidence-based implementation process including the following subprojects: “optimal pathways to prevention and care” [SP1], “improved institutional anchoring and professionals' attitudes, knowledge and skills” [SP2], “systematic screening, early detection, and family engagement” [SP3]. This systematical implementation process is developed to improve the implementation of prevention [SP4a] and therapy [SP4b].” SP5 realizes the online intervention i-chimps, and [SPs 6–8] realizes the medical, health economic, and qualitative evaluation [SPs 6–8]. In summary, ci-chimps realizes an overarching multicenter study to evaluate the implementation process with three implementation interventions [SP 1–3].

Ci-chimps also includes multiple interfaces with general medicine/primary care, pediatrics, youth healthcare services, and public sector services catering for the educational and residential needs of children. In the CHIMPS-NET model, the implementation of CHIMPS-P and CHIMPS-T in the model regions will be well-prepared through interventions at the interfaces, and in this way placed into an environment where experts from psychiatry and psychotherapy for children, adolescents, and adults as well as youth welfare and the civil sector are sensitized for the special requirements of those families. This implementation comprises all aspects of interdisciplinary and intersectional implementation, specific psychosocial intervention for children of parents with mental illness, quality assurance, cost-effectiveness, sustainability, and transfer.

### The Three Implementation Interventions in CHIMPS-NET

The main aim of ci-chimps is the evaluation of the effect of the three implementation interventions in CHIMPS-NET. Referring to this hierarchy, we deduce our three implementation interventions 1) optimal pathways to care, 2) education and training program for the professionals, and 3) systematic screening. To examine the utility of the supported implementation of CHIMPS-NET, a controlled trial design is used. With these three implementation interventions, we want to support the clinical implementation of the family-oriented interventions in CHIMPS-NET.

1) **Optimal pathways to care**

The first implementation measure focuses on optimal ways of caring for mentally ill patients who are also parents with children living in the household. The main goals of this intervention are the specific information of the referring physician, the indicated referral of mentally ill parents, and the consideration of underage children living in the household of the inpatient referral. The two main components are the evaluation of the attitudes of the medical and psychotherapeutic referrers toward the target group, and the intervention as well as the development and implementation of an optimal care pathway. First there is an analysis of the potential and actual referrer network. In the next step information is sent to these referrer network and in the last step there will be an evaluation and an follow-up analysis of the referrer network. The optimization of the allocation is organized by Silke Pawils (University Medical Center Hamburg-Eppendorf).

2) **Education and training program for the professionals**

The second implementation intervention concerns the improvement of the professionals' attitudes, knowledge, and skills. The two main components here being the assessment of the current state in all institutions within the clinical centers as well as the development and implementation of an education and training program. The employees of the randomized clinical centers in the intervention group will get a 3-h training. This intervention is organized by Svenja Taubner (University Hospital Heidelberg, Germany). Due to the Covid-19-pandemic, the training of medical staff is organized as two webinars. The first webinar contains knowledge about risks in mentally ill parents and intervention skills on how to address family-related problems with mentally ill parents or children, respectively. Herewith, a bi-focal perspective is demonstrated i.e., having the needs of parents on the one hand and of children on the other hand in mind when assessing needs and offering support. The second webinar is offered in an interactive format to discuss the content of the first webinar and specific implementation barriers between youth and adult psychiatric services as well as the practice of skills. Changes in attitudes toward working with mentally ill parents will be assessed over three times using the same online survey with the translated family-focused mental health practice questionnaire (26). The contents of the webinar have been published in more detail (27).

3) **Systematic screening**

The third implementation intervention includes a systematic screening process to improve the detection of mentally ill parents with affected children. Employees of the clinical centers fill out two short questionnaires with the parents. This intervention project is organized by Sibylle M. Winter (Charité, Berlin, Germany).

The first main question is whether psychiatric patients have responsibility for minors. If this question is answered in the affirmative, parents are presented with two short screening questionnaires of one page each. In the context of the study, sensitivity and specificity will be determined in comparison to standardized instruments (CBCL) and information collected during the family intervention.

The first questionnaire called “Children-Screening” (15) was developed specifically for children of mentally ill parents and piloted in the clinic for child and adolescent psychiatry of Charité. It records in short all potential mental health problems of a child. If the score is above the cut-off, further psychiatric assessment should be made. The second questionnaire called “Family-Screening” (15) was designed as a risk screening and records family risk and protective factors as well as incidences of domestic violence and neglect. The goal is to evaluate support needs for families. The result is presented in a traffic light system (RED-YELLOW-GREEN). RED indicates an urgent need for support. This questionnaire was developed in the child abuse clinic of the Charité and is also piloted there. Both questionnaires are not yet standardized, validated tools.

## Methods

### Aims and Hypotheses of the Study

In ci-chimps, we aim to determine if three implementation interventions are helpful in improving the clinical implementation of the CHIMPS-NET interventions. Additionally, we want to identify factors hindering or promoting implementation processes. In order to monitor the impact of the three implementation interventions, we will use the translated version of the “Family Focused Mental Health Practice Questionnaire (FFMHPQ)” (26) and the translated version of the “Implementation Components Questionnaire (ICQ)” (28). The introduction of these questionnaires in Germany has not yet been reported. So the aims of the study are 1) the cultural and linguistic adaptation of the “Family Focused Mental Health Practice Questionnaire” (26) and the “Implementation Components Questionnaire” (28) from English to German, 2) the first introduction of these questionnaires in Germany, 3) the evaluation of the effect of the three implementation interventions in CHIMPS-NET, and 4) the identification of factors which hinder or promote implementation processes of family-oriented interventions using the example of CHIMPS-NET intervention.

More specifically, the following hypotheses will be tested:

H1: In this randomized controlled multicenter trial, we will compare the family-oriented practice between the clinical centers receiving the support of the three implementation interventions and the clinical centers not receiving the support of the implementation interventions. Our primary hypothesis is that clinical centers receiving at least one of the implementation interventions work more family-oriented than clinical centers without the support. Higher values after 12- and 24-months follow-up compared to the baseline mean a higher family-oriented practice. This is measured with the translated version of the “Family Focused Mental Health Practice Questionnaire.”
H2: Furthermore, with our secondary hypothesis, we will compare the personnel's satisfaction regarding the implementation of the CHIMPS-NET project between the clinical centers receiving additionally the support of the three implementation interventions and the clinical centers not receiving the support of the implementation interventions. The personnel's satisfaction regarding the implementation of the CHIMPS-NET project is higher in the clinical centers receiving the implementation interventions than the personnel's satisfaction in the clinical centers without the three implementation interventions. This is measured with the translated version of the “Implementation Components Questionnaire” where higher values mean higher personnel satisfaction.

### Study Design

**Figure 2:**
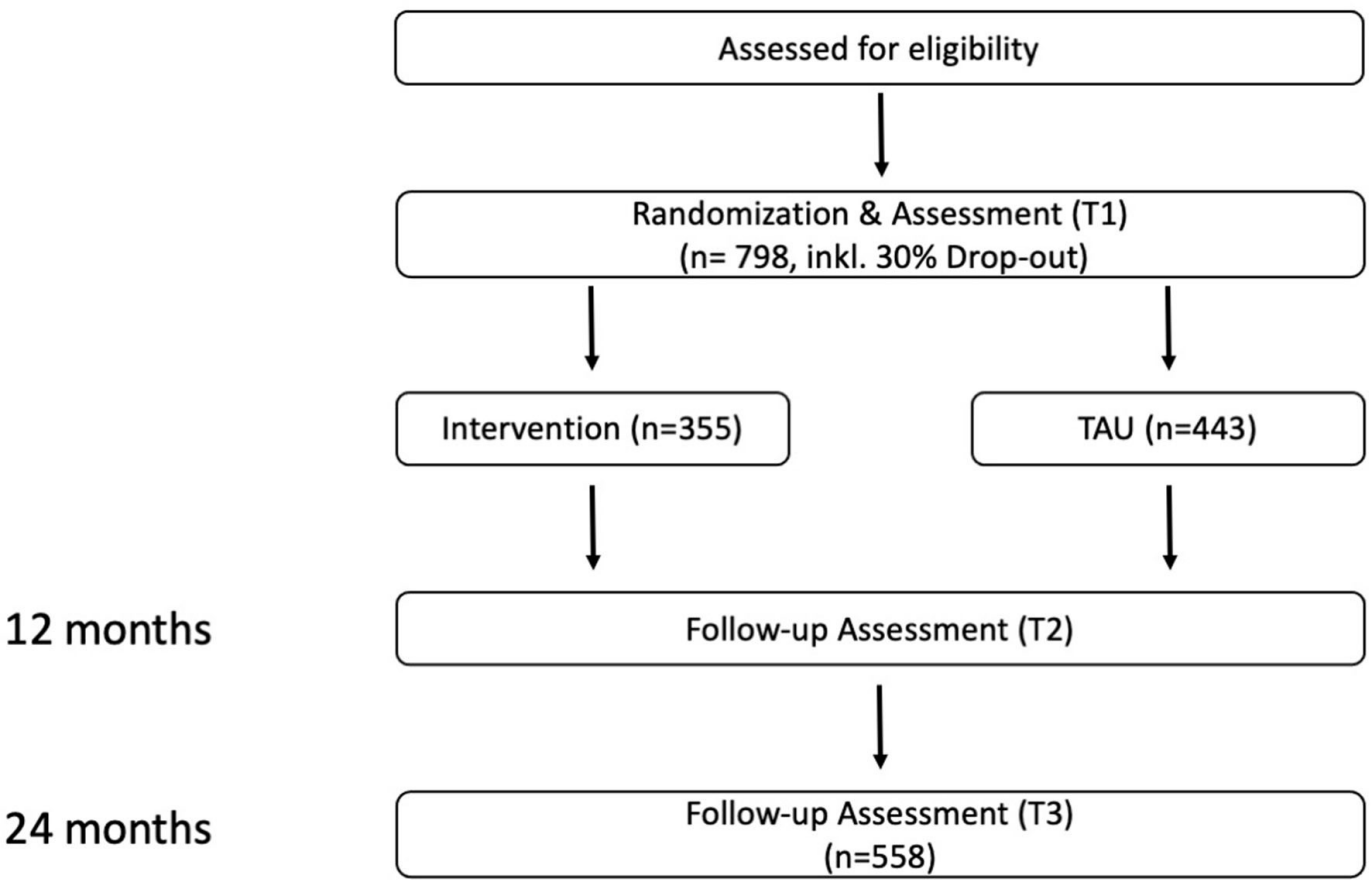
Study design.

As you can see in Figure 2 the ci-chimps study is a two-group randomized controlled multicenter trial with assessments at baseline as well as at 12- and 24-months follow-up. It is one part of the superior project CHIMPS-NET, wherefore an own study protocol is in preparation (9). The ci-chimps study will be conducted from January 2020 to September 2023. The ci-chimps project's preparation phase includes the translation of the questionnaires FFMHPQ and the ICQ and the randomization of the clinical centers. The first measurement (baseline) takes place from January 2020 to May 2021. We stopped the baseline assessment after the last implementation intervention and defined this point as the end of the implementation interventions. During this year we implemented the superior project CHIMPS and the three implementation interventions of ci-chimps in the clinical centers. The second assessment will be in May 2022 and the follow-up in May 2023.

### Study Setting

At the beginning of the study, 21 clinical centers located in 15 federal states in Germany were part of the CHIMPS-NETwork. Each clinical center has two subordinate clinics: one of the children and adolescent psychiatric department and one of the adult psychiatric department. Only 19 of the 21 clinical centers were randomized such that nine centers will receive additional support of all of the three implementation interventions (intervention group), and the remaining 10 clinical centers will be the control group and will not get specific implementation support. Hamburg (University Medical Centre Hamburg-Eppendorf, Germany), as the headquarter, and Munich (University Medical Center, Department of Psychiatry and Psychotherapy, Munich, Germany) are conducting their own study) are not randomized. Unfortunately, one clinical center of the intervention group left the study after a few weeks, so eight clinical centers remain in the intervention group and 10 in the control group. Because we defined the number of cases per clinical center, the unequal allocation to the intervention and control group leads to the consequence that the number of cases is different for the intervention- and the control group (TAU) as seen in Figure 1.

### Participants

#### Sample Size and Power Calculation

This study will be conducted in 18 clinical centers and 31 employees on average per clinical center will be required to detect a difference at 12 and 24 months follow-up regarding family-oriented practice measured with FFMHPQ between the clinical centers receiving the support of the three implementation interventions and the clinical centers not receiving the support of the implementation interventions of 0.4 (Cohen's d) with 80% power, a two-sided alpha of 5% and a cluster effect of 5% (intraclass correlation coefficient of 0.05). This results in a sample size of 558 employees (31 per clinical center). Assuming a proportion of 30% incompletely completed questionnaires, we aim to recruit 798 employees.

#### Randomization

The random allocation of the clinical centers that receive or do not receive the support in a ratio of 1:1 was conducted according to a central randomization list generated with the statistical software R version 3.6.3 by the Department of Medical Biometry and Epidemiology (University Medical Centre Hamburg-Eppendorf, Germany) outside the recruitment and clinic. Originally, 18 centers participated in the study. After the randomization, another center was recruited. This center was randomized individually. At the end of this procedure, one center withdrew its participation in the study. So, we have an allocation ratio of 8:10.

#### Criteria for Inclusion

Every employee involved in the treatment of the patients (Medicine, Psychology, Nurses) of every clinical center that is part of the CHIMPS-NET project will be invited to participate in the ci-chimps study.

#### Criteria for Exclusion

There are no explicit criteria for exclusion.

### Outcome Measures

#### Sociodemographic Questions

Besides age, gender and profession, we record with a specifically designed questionnaire, in which clinical center the employees work, how long they have been employed there and how strongly they are involved in the CHIMPS-NET-project.

#### Family Focused Mental Health Practice Questionnaire (FFMHPQ)

The “Family Focused Mental Health Practice Questionnaire (FFMHPQ)” (29) has 18 subscales, comprising a total of 53 items and measures numerous aspects relevant for family-oriented practice from the employee's point of view, on a 7-point Likert scale (ranging from strongly disagree to strongly agree plus “not applicable”). In addition to organizational and political aspects (e.g., workplace support, guidelines, local conditions, workload), the questionnaire determines the needs of hospital employees (e.g., knowledge transfer, skills about family issues, their interest in working with children, parents, and families) and families (e.g., psychoeducation). It also takes into account “external” factors such as the general organization of the health system and geographical conditions, as well as socio-demographic data. The Cronbach's alpha coefficients of the FFMHPQ range from 0.70 to 0.90 for most subscales. The FFMHPQ has demonstrated excellent face and content validities (29).

#### Implementation Components Questionnaire (ICQ-35)

In ci-chimps, we are using the shorter version of the “Implementation Components Questionnaire (ICQ-35)" (30). The 35 items questionnaire has nine subscales with five choices of response: “not applicable, yes, sometimes, no, don't know.” The employees are asked to rate their perceived level of integration of the intervention within their clinical center. It determines special components of implementation, like selection or training of the employees. The Cronbach's alphas of the subscales range from 0.67 to 0.83. The original long version of the ICQ has 89 items and was first adapted in Norway (31, 32) from an earlier version of the Measures of Implementation Components of the National Implementation Research Network Frameworks by Ogden et al. (31) and Fixsen et al. (32). It has been shown to have good psychometric validity (32).

#### Implementation Satisfaction Scale (ISS)

Additionally, we are using the 4 item “Implementation Satisfaction Scale (ISS)” (30) which is part of the ICQ. It measures how satisfied the employees are with the implementation process on a 5-point Likert scale (ranging from strongly disagree to strongly agree). The Cronbach's alpha of the implementation satisfaction scale is 0.88 (30).

### Translation Procedure

Up to now, the FFMHPQ has been used in different settings (e.g., family helplines, adult psychiatry) in various occupational groups (e.g., nurses, doctors) in Australia, Portugal, Thailand, Japan, Ireland, and Norway. The introduction of this questionnaire in Germany is still missing. The same applies to the ICQ and ISS. Adapting the FFMHPQ, the ICQ and the ISS to Germany should enable more effective implementation of family-oriented interventions in the regular healthcare system in the future.

According to the cross-cultural translation procedure recommended by Beaton and Guillemin (33), the questionnaires are translated from English to German and back into English. Two translators with German as their first language independently translate the questionnaire from English to German. Both versions (T1 and T2) are compared, differences are discussed and a third combined version T12 (synthesis from both translations) is developed. In the next step, two translators with English as their first language translate the questionnaire version T12 from German back into English (BT1 and BT2). Subsequently, the original questionnaires, the versions T1, T2, T12, BT1, and BT2 as well as the interim reports are discussed and evaluated within the framework of an expert committee. As a final step in the adaptation process, clinic employees are asked how they interpret the items and evaluate the questionnaire (applicability test). All questionnaires were slightly modified or reworded to adapt them to our target group.

### Statistical Analysis

#### Data Coding and Analysis

All statistical analyses will be performed with SPSS (version 26.0 or newer). Means and standard deviations or median and 1st and 3rd quartiles, as appropriate, for the continuous variables as well as absolute and relative frequencies for the categorical variables of the whole sample and the respective treatment groups will be calculated and presented. The primary analysis will be conducted with the intention to treat (ITT) population consisting of all employees. The employees of a clinical center are subject to the same influences and cluster effects. Hence, for the primary endpoint, operationalized as the difference to baseline, a mixed linear model will be performed with treatment group, time, and baseline value as fixed effects, and clinical center as a random effect. The interaction between the treatment group and time will be tested and will be eliminated from the model if it is not statistically significant. The result of the primary analysis is the contrast of the treatment group after 18 months. Only this result will be considered in a confirmatory manner. The two-sided type I error will be set to 5%. It is possible that the fluctuation of employees in the clinical centers is so low that we can extend the model to a longitudinal model. The secondary endpoints will be examined in an exploratory manner with the same model in the case of continuous endpoints and with a similarly mixed Poisson regression in the case of count data. Results will be reported and published according to the CONSORT statement for cluster randomized trials (34).

## Discussion

While research has identified good evidence-based family-oriented interventions like CHIMPS that improve the quality of life of families, little is known about how to implement such interventions most effectively and efficiently in clinical practice in Germany. A strength of this ci-chimps -study is the use of a randomized controlled multicenter trial design with a large and wide-ranging sample. We are including both the child and adolescent as well as adult psychiatric departments and all professionals within a clinical center to get a better understanding of the implementation barriers. It could be a limitation that we can't ensure that the same employees fill out all four measurement times. For the first time in Germany, the translated versions of the questionnaires FFMHPQ and ICQ are used in Germany, so there is no German psychometric data yet. However, both original versions have demonstrated good psychometric validities in previous studies. Thus, it can be assumed that the translated German versions will show good psychometric validities as well.

Generally, it should be considered that ci-chimps is a purely questionnaire-based study, relying on the principle of self-disclosure. Therefore, the tendency toward social desirability cannot be ruled out. However, it can be assumed that this potential trend does not significantly affect the results, because the focus is on the comparison between employees from different clinical centers and the probability is high that this tendency can be found for both groups.

With ci-chimps, the implementation of CHIMPS is being supported for the first time. In previous studies of CHIMPS, there were no specific implementation interventions. Therefore, it should be considered that we can only measure an overall intervention effect. It is not possible to compare the three implementation interventions amongst each other. However, we are sure that with ci-chimps we will be able to identify training needs in clinics in order to improve the implementation of family-oriented interventions like CHIMPS-NET in the future.

## Ethics Statement

The studies involving human participants were reviewed and approved by Ethik Kommission der Ärztekammer Hamburg. The patients/participants provided their written informed consent to participate in this study.

## Author Contributions

CL and AM are responsible for the data and implementation study management. SW-G is the principal researcher of this implementation study ci-chimps, in cooperation with DM, AR, and BS, and of the whole CHIMPS-NET. CL drafted the manuscript with the help of SW-G, DM, and BS. SW-G, DM, and AR were substantially involved in the conception of the study and contributed to its design. AD, AH, and AZ are supported by the paragraphs on statistical methods and the randomization of the clinical centers. SP, ST, and SW realized as the project leaders the three implementation interventions. All authors have participated in the editing of the manuscript and have read and approved the final manuscript.

## Funding

The study described in this protocol was funded by the German Federal Joint Committee (G-BA).

## Conflict of Interest

The authors declare that the research was conducted in the absence of any commercial or financial relationships that could be construed as a potential conflict of interest.

## Publisher's Note

All claims expressed in this article are solely those of the authors and do not necessarily represent those of their affiliated organizations, or those of the publisher, the editors and the reviewers. Any product that may be evaluated in this article, or claim that may be made by its manufacturer, is not guaranteed or endorsed by the publisher.

## Abbreviations

BMBF: bundesministerium für bildung und forschung (federal ministry of education and research)
CHIMPS: children of mentally ill parents
CHIMPS-NET: children of mentally ill parents research network
CHIMPS-P: CHIMPS-prevention (families with noticeable problems but no own diagnosis yet)
CHIMPS-T: CHIMPS-therapy
Ci-chimps: the clinical implementation study of CHIMPS with three implementation interventions
FFMHPQ: family focused mental health practice questionnaire
GB-A: gemeinsamer bundesausschuss (joint federal committee)
ICQ: implementation components questionnaire
ISS: implementation satisfaction scale
SPSS: statistical package for the social sciences.
